# Comparative Analysis of Deterministic and Nondeterministic Decision Trees for Decision Tables from Closed Classes

**DOI:** 10.3390/e26060519

**Published:** 2024-06-17

**Authors:** Azimkhon Ostonov, Mikhail Moshkov

**Affiliations:** Computer, Electrical and Mathematical Sciences & Engineering Division and Computational Bioscience Research Center, King Abdullah University of Science and Technology (KAUST), Thuwal 23955-6900, Saudi Arabia; mikhail.moshkov@kaust.edu.sa

**Keywords:** closed classes of decision tables, deterministic decision trees, nondeterministic decision trees

## Abstract

In this paper, we consider classes of decision tables with many-valued decisions closed under operations of the removal of columns, the changing of decisions, the permutation of columns, and the duplication of columns. We study relationships among three parameters of these tables: the complexity of a decision table (if we consider the depth of the decision trees, then the complexity of a decision table is the number of columns in it), the minimum complexity of a deterministic decision tree, and the minimum complexity of a nondeterministic decision tree. We consider the rough classification of functions characterizing relationships and enumerate all possible seven types of relationships.

## 1. Introduction

In this paper, we consider closed classes of decision tables with many-valued decisions and study the relationships among three parameters of these tables: the complexity of a decision table (if we consider the depth of decision trees, then the complexity of a decision table is the number of columns in it), the minimum complexity of a deterministic decision tree, and the minimum complexity of a nondeterministic decision tree.

A decision table with many-valued decisions is a rectangular table in which columns are labeled with attributes, rows are pairwise different, and each row is labeled with a nonempty, finite set of decisions. Rows are interpreted as tuples of values of the attributes. For a given row, it is required to find a decision from the set of decisions attached to the row. To this end, we can use the following queries: we can choose an attribute and ask what is the value of this attribute in the considered row. We study two types of algorithms based on these queries: deterministic and nondeterministic decision trees. One can interpret nondeterministic decision trees for a decision table as a way to represent an arbitrary system of true decision rules for this table that covers all rows. We consider in some sense arbitrary complexity measures that characterize the time complexity of decision trees. Among them, we distinguish so-called limited complexity measures, for example, the depth of decision trees.

Decision tables with many-valued decisions often appear in data analysis, where they are known as multilabel decision tables [1,2,3]. Moreover, decision tables with many-valued decisions are common in such areas as combinatorial optimization, computational geometry, and fault diagnosis, where they are used to represent and explore problems.

Decision trees [4,5,6,7] and decision rule systems [8,9,10,11,12] are widely used as classifiers as a means for knowledge representation and as algorithms for solving various problems of combinatorial optimization, fault diagnosis, etc. Decision trees and rules are among the most interpretable models in data analysis [13].

The depth of deterministic and nondeterministic decision trees for computation Boolean functions (variables of a function are considered as attributes) has been studied quite intensively [14,15,16]. Note that the minimum depth of a nondeterministic decision tree for a Boolean function is equal to its certificate complexity [17].

We study classes of decision tables with many-valued decisions closed under four operations: the removal of columns, the changing of decisions, the permutation of columns, and the duplication of columns. The most natural examples of such classes are closed classes of decision tables generated by information systems [18]. An information system consists of a set of objects (universe) and a set of attributes (functions) defined on the universe and with values from a finite set. A problem over an information system is specified by a finite number of attributes that divide the universe into nonempty domains in which these attributes have fixed values. A nonempty finite set of decisions is attached to each domain. For a given object from the universe, it is required to find a decision from the set attached to the domain containing this object.

A decision table with many-valued decisions corresponds to this problem in a natural way: the columns of this table are labeled with the considered attributes, and the rows correspond to domains and are labeled with sets of decisions attached to domains. The set of decision tables corresponding to problems over an information system forms a closed class generated by this system. Note that the family of all closed classes is essentially wider than the family of closed classes generated by information systems. In particular, the union of two closed classes generated by two information systems is a closed class. However, generally, there is not an information system that generates this class.

Various classes of objects that are closed under different operations have been intensively studied. Among them, in particular, are classes of Boolean functions closed under the operation of superposition [19], minor-closed classes of graphs [20], classes of read-once Boolean functions closed under the removal of variables and the renaming of variables, languages closed under taking factors, etc. Decision tables represent an interesting mathematical object deserving mathematical research, particularly regarding the study of closed classes of decision tables.

This paper continues the study of closed classes of decision tables that started with the work of [21] and that were frozen for various reasons for many years. In [21], we studied the dependence of the minimum depth of deterministic decision trees and the depth of deterministic decision trees constructed by a greedy algorithm on the number of attributes (columns) for conventional decision tables from classes closed under operations of the removal of columns and the changing of decisions.

In the present paper, we study so-called t pairs (C,ψ), where C is a class of decision tables closed under the considered four operations, and ψ is a complexity measure for this class. The t pair is called limited if ψ is a limited complexity measure. For any decision table T∈C, we have three parameters:

- $\psi^{i}\left(T\right)$—The complexity of the decision table *T*. This parameter is equal to the complexity of a deterministic decision tree for the table *T*, which sequentially computes the values of all attributes attached to columns of *T*.
- $\psi^{d}\left(T\right)$—The minimum complexity of a deterministic decision tree for the table *T*.
- $\psi^{a}\left(T\right)$—The minimum complexity of a nondeterministic decision tree for the table *T*.

We investigate the relationships between any two such parameters for decision tables from C. Let us consider, for example, the parameters $\psi^{i}\left(T\right)$ and $\psi^{d}\left(T\right)$. Let n∈N. We study relations of the kind $\psi^{i}\left(T\right)\leq n\Rightarrow \psi^{d}\left(T\right)\leq u$, which are true for any table T∈C. The minimum value of *u* is the most interesting for us. This value (if it exists) is equal to

$$U_{C\psi }^{di}\left(n\right)=\max\left\{\psi^{d}\left(T\right):T\in C,\psi^{i}\left(T\right)\leq n\right\}.$$

We also study relations of the kind $\psi^{i}\left(T\right)\geq n\Rightarrow \psi^{d}\left(T\right)\geq l$. In this case, the maximum value of *l* is the most interesting for us. This value (if it exists) is equal to

$$L_{C\psi }^{di}\left(n\right)=\min\left\{\psi^{d}\left(T\right):T\in C,\psi^{i}\left(T\right)\geq n\right\}.$$

The two functions $U_{C\psi }^{di}$ and $L_{C\psi }^{di}$ describe how the behavior of the parameter $\psi^{d}\left(T\right)$ depends on the behavior of the parameter $\psi^{i}\left(T\right)$ for tables from C.

There are 18 similar functions for all ordered pairs of parameters $\psi^{i}\left(T\right)$, $\psi^{d}\left(T\right)$, and $\psi^{a}\left(T\right)$. These 18 functions well describe the relationships among the considered parameters. It would be very interesting to point out the 18 tuples of these functions for all t pairs and all limited t pairs. But, this is a very difficult problem.

In this paper, instead of functions, we study types of functions. With any partial function f:N→N, we associate its type from the set {α,β,γ,δ,ϵ}. For example, if the function *f* has an infinite domain, and it is bounded from above, then its type is equal to α. If the function *f* has an infinite domain, is not bounded from above, and the inequality f(n)≥n holds for a finite number of n∈N, then its type is equal to β. Thus, we enumerate the 18 tuples of the types of functions. These tuples are represented in tables called the types of t-pairs. We prove that there are only seven realizable types of t pairs and only five realizable types of limited t pairs.

First, we study 9 tuples of the types of functions $U_{C\psi }^{bc}$, b,c∈{i,d,a}. These tuples are represented in tables called upper types of t pairs. We enumerate all the realizable upper types of t pairs and limited t pairs. After that, we extend the results obtained for the upper types of t pairs to the case of the types of t pairs. We also define the notion of a union of two t pairs and study the upper type of the resulting t pair, thus depending on the upper types of the initial t pairs.

The obtained results allow us to point out cases where the complexity of deterministic and nondeterministic decision trees is essentially less than the complexity of the decision table (see Section 2.3). This finding may prove useful in related applications.

This paper is based on the work of [22], in which similar results were obtained for classes of problems over information systems. We have generalized proofs from [22] to the case of decision tables from closed classes and use some results from this paper to prove the existence of t pairs and limited t pairs with given upper types.

In our previous work [7], we considered functions characterizing the growth in the worst case of the minimum complexity of deterministic and nondeterministic decision trees with the growth of the complexity of the set of attributes attached to columns of the conventional decision table and also obtained preliminary results on the behavior of the function characterizing the relationship between the former two parameters. In the current work, we mainly focus on the rough classification of types.

The paper consists of eight sections. In Section 2, the basic definitions are considered. In Section 3, we provide the main results related to the types of t pairs and limited t pairs. In Section 4, Section 5 and Section 6, we study the upper types of t pairs and the limited t pairs. Section 7 contains proofs of the main results, and Section 8 provides short conclusions.

## 2. Basic Definitions

### 2.1. Decision Tables and Closed Classes

Let N={0,1,2,…} be the set of non-negative integers. For any k∈N∖{0,1}, let $E_{k}=\left\{0,1,\ldots ,k-1\right\}$. The set of nonempty finite subsets of the set N will be denoted by P(N). Let *F* be a nonempty set of *attributes* (really, the names of attributes).

**Definition** **1.** *We now define the set of decision tables $M_{k}\left(F\right)$. An arbitrary decision table T from this set is a rectangular table with n∈N∖{0} columns labeled with attributes $f_{1},\ldots ,f_{n}\in F$, where any two columns labeled with the same attribute are equal. The rows of this table are pairwise different and are filled in with numbers from $E_{k}$. Each row is interpreted as a tuple of values of attributes $f_{1},\ldots ,f_{n}$. For each row in the table, a set from P(N) is attached, which is interpreted as a set of decisions for this row.*

**Figure 1:**
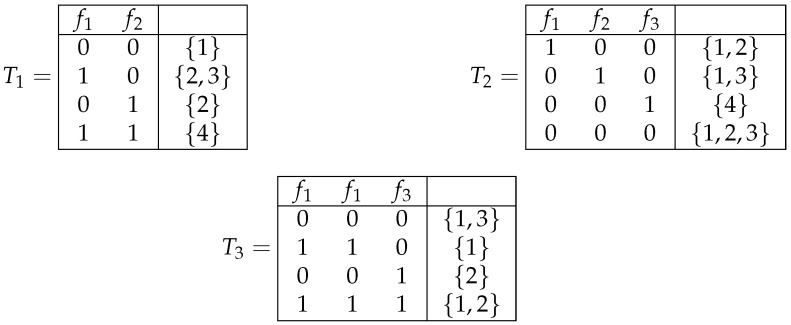
Decision tables $T_{1}$, $T_{2}$, and $T_{3}$.

**Example** **1.** *Three decision tables $T_{1}$, $T_{2}$, and $T_{3}$ from the set $M_{2}\left(F_{0}\right)$, where $F_{0}=\left\{f_{1},f_{2},f_{3}\right\}$, are shown in Figure 1.*

We correspond to the table *T* the following *problem*: for a given row of *T*, we should recognize a decision from the set of decisions attached to this row. To this end, we can use queries about the values of the attributes for this row.

We denote as At(T) the set $\left\{f_{1},\ldots ,f_{n}\right\}$ of attributes attached to the columns of *T*. Π(T) denotes the intersection of the sets of decisions attached to the rows of *T*, and by Δ(T), we denote the set of rows of the table *T*. Decisions from Π(T) are called *common decisions* for *T*. The table *T* will be called *degenerate* if Δ(T)=⌀ or if Π(T)≠⌀. We denote as $M_{k}^{c}\left(F\right)$ the set of degenerate decision tables from $M_{k}\left(F\right)$.

**Figure 2:**
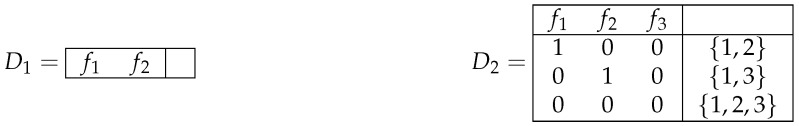
Degenerate decision tables $D_{1}$ and $D_{2}$.

**Example** **2.** *Two degenerate decision tables, $D_{1}$ and $D_{2}$, are shown in Figure 2.*

**Definition** **2.** A subtable of the table T is a table obtained from T through the removal of some of its rows. Let $\Theta \left(T\right)=\{\left(f,\delta \right):f\in At\left(T\right),\delta \in E_{k}\}$ and $\Theta^{*}\left(T\right)$ be the set of all finite words in the alphabet Θ(T), including the empty word λ. Let $\alpha \in \Theta^{*}\left(T\right)$. We now define a subtable Tα of the table T. If α=λ, then Tα=T. Let $\alpha =\left(f_{i_{1}},\delta_{1}\right)\cdots \left(f_{i_{m}},\delta_{m}\right)$. Then, Tα consists of all the rows of T that, in the intersection with columns $f_{i_{1}},\ldots ,f_{i_{m}}$, have values $\delta_{1},\ldots ,\delta_{m}$, respectively.

**Figure 3:**
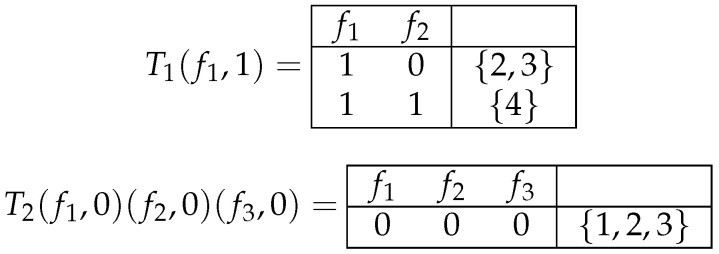
Subtables $T_{1}\left(f_{1},1\right)$ and $T_{2}\left(f_{1},0\right)\left(f_{2},0\right)\left(f_{3},0\right)$ of tables $T_{1}$ and $T_{2}$ shown in Figure 1.

**Example** **3.** *Two subtables of the tables $T_{1}$ and $T_{2}$ (depicted in Figure 1) are shown in Figure 3.*

We now define four operations on the set $M_{k}\left(F\right)$ of decision tables:

**Definition** **3.** *Removal of columns: We can remove an arbitrary column in a table T with at least two columns. As a result, the obtained table can have groups of equal rows. We keep only the first row in each such group.*

**Definition** **4.** *Changing of decisions: In a given table T, we can change in an arbitrary way sets of decisions attached to rows.*

**Definition** **5.** *Permutation of columns: We can swap any two columns in a table T, including the attached attribute names.*

**Definition** **6.** *Duplication of columns: For any column in a table T, we can add its duplicate next to that column.*

Definitions 5 and 6 characterize the two most natural examples of operations applied to information systems. Definitions 3 and 4 allows us to say that we cover important classes of information systems (see Section 2.4).

**Figure 4:**
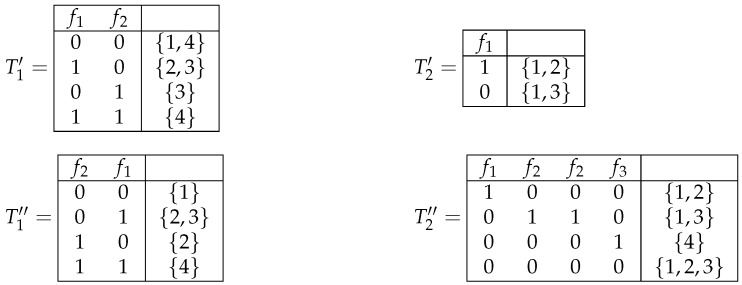
Decision tables $T_{1}^{\prime }$,$T_{2}^{\prime }$,$T_{1}^{\prime \prime }$, and $T_{2}^{\prime \prime }$ obtained from tables $T_{1}$ and $T_{2}$ shown in Figure 1 by operations of changing the decisions, removal of columns, permutation of columns, and duplication of columns, respectively.

**Example** **4.** *Decision tables $T_{1}^{\prime }$,$T_{2}^{\prime }$,$T_{1}^{\prime \prime }$, and $T_{2}^{\prime \prime }$ depicted in Figure 4 are obtained from decision tables $T_{1}$ and $T_{2}$ shown in Figure 1 by operations of changing the decisions, removal of columns, permutation of columns, and duplication of columns, respectively.*

**Definition** **7.** *Let $T\in M_{k}\left(F\right)$. The closure of the table T is a set, which contains all the tables that can be obtained from T by the operations of the removal of columns, the changing of decisions, the permutation of columns, and the duplication of columns using only such tables. We denote the closure of the table T by [T]. It is clear that T∈[T].*

**Definition** **8.** *Let $C\subseteq M_{k}\left(F\right)$. The closure [C] of the set C is defined in the following way: $\left[C\right]={⋃}_{T\in C}\left[T\right]$. We say that C is a closed class if C=[C]. In particular, the empty set of tables is a closed class.*

**Figure 5:**
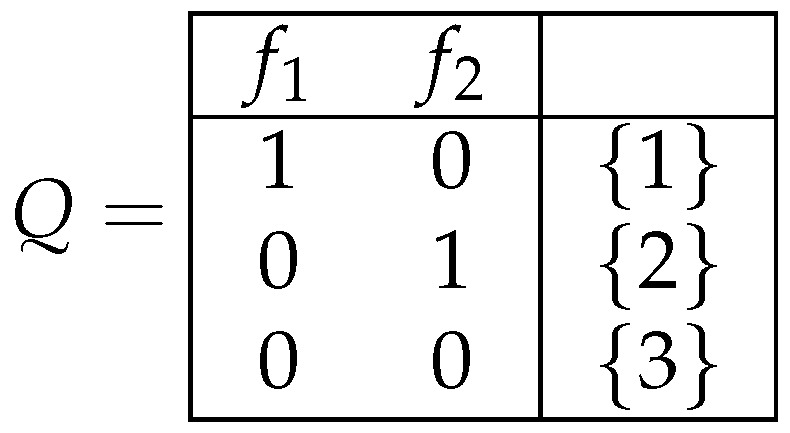
Decision table *Q*.

**Figure 6:**
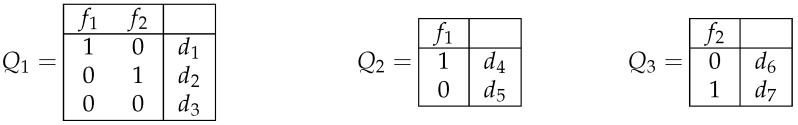
Decision tables from closed class $C_{0}$, where $d_{1},\ldots ,d_{7}\in P\left(N\right)$.

**Example** **5.** *We now consider a closed class $C_{0}$ of decision tables from the set $M_{2}\left(\left\{f_{1},f_{2}\right\}\right)$, which is equal to [Q], where the decision table Q is depicted in Figure 5. The closed class $C_{0}$ contains all the tables depicted in Figure 6 and all the tables that can be obtained from them by the operations of the duplication of columns and the permutation of columns.*

If $C_{1}$ and $C_{2}$ are closed classes belonging to $M_{k}\left(F\right)$, then $C_{1}\cup C_{2}$ is also a closed class. We can consider closed classes $C_{1}$ and $C_{2}$ belonging to different sets of decision tables. Let $C_{1}\subseteq M_{k_{1}}\left(F_{1}\right)$ and $C_{2}\subseteq M_{k_{2}}\left(F_{2}\right)$. Then, $C_{1}\cup C_{2}$ is a closed class, and $C_{1}\cup C_{2}\subseteq M_{\max(k_{1},k_{2})}\left(F_{1}\cup F_{2}\right)$.

### 2.2. Deterministic and Nondeterministic Decision Trees

A *finite directed tree with the root* is a finite directed tree in which exactly one node has no entering edges. This node is called the *root*. Nodes of the tree, which have no outgoing edges, are called *terminal* nodes. Nodes that are neither the root nor the terminal are called *worker* nodes. A *complete path* in a finite directed tree with the root is any sequence of nodes and edges starting from the root node and ending with a terminal node $\xi =v_{0},d_{0},\ldots ,v_{m},d_{m},v_{m+1}$, where $d_{i}$ is the edge outgoing from the node $v_{i}$ and entering the node $v_{i+1},i=0,\ldots ,m$.

**Definition** **9.** *A decision tree over the set of decision tables $M_{k}\left(F\right)$ is a labeled finite directed tree with the root with at least two nodes (the root and a terminal node) possessing the following properties:*

• *The root and the edges outgoing from the root are not labeled.*
• *Each worker node is labeled with an attribute from the set F.*
• *Each edge outgoing from a worker node is labeled with a number from $E_{k}$.*
• *Each terminal node is labeled with a number from N.*

We denote as $T_{k}\left(F\right)$ the set of decision trees over the set of decision tables $M_{k}\left(F\right)$.

**Definition** **10.** *A decision tree from $T_{k}\left(F\right)$ is called deterministic if it satisfies the following conditions:*

• *Exactly one edge leaves the root.*
• *The edges outgoing from each worker node are labeled with pairwise different numbers.*

Let Γ be a decision tree from $T_{k}\left(F\right)$. Denote as At(Γ) the set of attributes attached to the worker nodes of Γ. Set $\Theta \left(\Gamma \right)=\{\left(f,\delta \right):f\in At\left(\Gamma \right),\delta \in E_{k}\}$. Denote as $\Theta^{*}\left(\Gamma \right)$ the set of all finite words in the alphabet Θ(Γ), including the empty word λ. We correspond to an arbitrary complete path $\xi =v_{0},d_{0},\ldots ,v_{m},d_{m},v_{m+1}$ in Γ, as well as a word π(ξ). If m=0, then π(ξ)=λ. Let m>0 and, for i=1,…,m, the node $v_{i}$ is labeled with an attribute $f_{j_{i}}$, and the edge $d_{i}$ is labeled with the number $\delta_{i}$. Then, $\pi \left(\xi \right)=\left(f_{j_{1}},\delta_{1}\right)\cdots \left(f_{j_{m}},\delta_{m}\right)$. We denote as τ(ξ) the number attached to the terminal node of the path ξ. We denote as Path(Γ) the set of complete paths in the tree Γ.

**Definition** **11.** *Let $T\in M_{k}\left(F\right)$. A nondeterministic decision tree for the table T is a decision tree *Γ* over $M_{k}\left(F\right)$ satisfying the following conditions:*

-
At(Γ)⊆At(T).

-
${⋃}_{\xi \in Path(\Gamma )}\Delta \left(T\pi \left(\xi \right)\right)=\Delta \left(T\right).$

- *For any row r∈Δ(T) and any complete path ξ∈Path(Γ), if r∈Δ(Tπ(ξ)), then τ(ξ) belongs to the set of decisions attached to the row r.*

**Figure 7:**
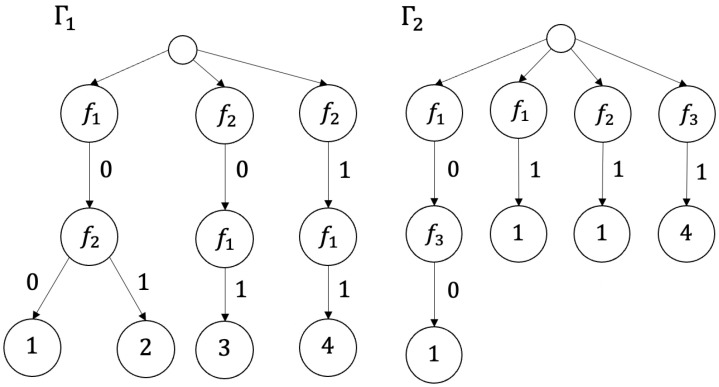
Nondeterministic decision trees $\Gamma_{1}$ and $\Gamma_{2}$ for decision tables $T_{1}$ and $T_{2}$ depicted in Figure 1.

**Example** **6.** *Nondeterministic decision trees $\Gamma_{1}$ and $\Gamma_{2}$ for decision tables $T_{1}$ and $T_{2}$ shown in Figure 1 are depicted in Figure 7.*

**Definition** **12.** *A deterministic decision tree for the table T is a deterministic decision tree over $M_{k}\left(F\right)$, which is a nondeterministic decision tree for the table T.*

**Figure 8:**
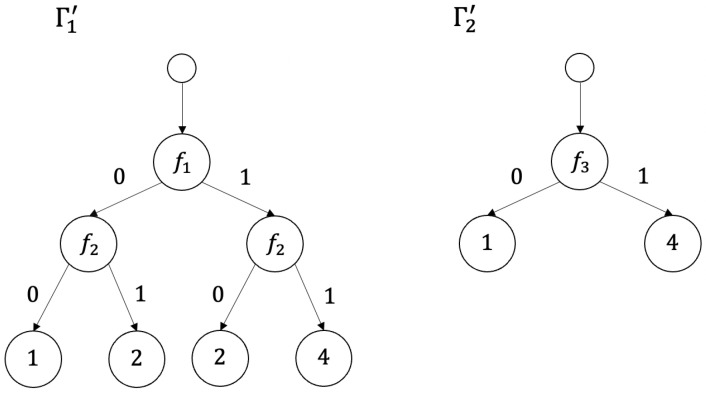
Deterministic decision trees $\Gamma_{1}^{\prime }$ and $\Gamma_{2}^{\prime }$ for decision tables $T_{1}$ and $T_{2}$ depicted in Figure 1.

**Example** **7.** *Deterministic decision trees $\Gamma_{1}^{\prime }$ and $\Gamma_{2}^{\prime }$ for decision tables $T_{1}$ and $T_{2}$ shown in Figure 1 are depicted in Figure 8.*

### 2.3. Complexity Measures

Denote as $F^{*}$ the set of all finite words over the alphabet *F*, including the empty word λ.

**Definition** **13.** A complexity measure over the set of decision tables $M_{k}\left(F\right)$ is any mapping $\psi :F^{*}\to N$.

**Definition** **14.** *The complexity measure ψ will be called limited if it possesses the following properties:*

**(a)**  $\psi \left(\alpha_{1}\alpha_{2}\right)\leq \psi \left(\alpha_{1}\right)+\psi \left(\alpha_{2}\right)$ for any $\alpha_{1},\alpha_{2}\in F^{*}$.
**(b)**  $\psi \left(\alpha_{1}\alpha_{2}\alpha_{3}\right)\geq \psi \left(\alpha_{1}\alpha_{3}\right)$ for any $\alpha_{1},\alpha_{2},\alpha_{3}\in F^{*}$.
**(c)**  For any $\alpha \in F^{*}$, the inequality ψ(α)≥|α| holds, where |α| is the length of α.

We extend an arbitrary complexity measure ψ onto the set $T_{k}\left(F\right)$ in the following way. Let $\Gamma \in T_{k}\left(F\right)$. Then, ψ(Γ)=max{ψ(φ(ξ)):ξ∈Path(Γ)}, where φ(ξ)=λ if π(ξ)=λ and $\varphi \left(\xi \right)=f_{1}\cdots f_{m}$ if $\pi \left(\xi \right)=\left(f_{1},\delta_{1}\right)\cdots \left(f_{m},\delta_{m}\right)$. The value ψ(Γ) will be called the *complexity of the decision tree* Γ.

We now consider an example of a complexity measure. Let w:F→N∖{0}. We define the function $\psi^{w}:F^{*}\to N$ in the following way: $\psi^{w}\left(\alpha \right)=0$ if α=λ and $\psi^{w}\left(\alpha \right)=\sum_{i=1}^{m}w\left(f_{i}\right)$ if $\alpha =f_{1}\cdots f_{m}$. The function $\psi^{w}$ is a limited complexity measure over $M_{k}\left(F\right)$, and it is called a *weighted depth*. If w≡1, then the function $\psi^{w}$ is called the *depth* and is denoted by *h*.

Let ψ be a complexity measure over $M_{k}\left(F\right)$ and *T* be a decision table from $M_{k}\left(F\right)$, in which rows are labeled with attributes $f_{1},\ldots ,f_{n}$. The value $\psi^{i}\left(T\right)=\psi \left(f_{1}\cdots f_{n}\right)$ is called the *complexity of the decision table T*. We denote by $\psi^{d}\left(T\right)$ the minimum complexity of a deterministic decision tree for the table *T*. We denote by $\psi^{a}\left(T\right)$ the minimum complexity of a nondeterministic decision tree for the table *T*.

### 2.4. Information Systems

Let *A* be a nonempty set and *F* be a nonempty set of functions from *A* to $E_{k}$.

**Definition** **15.** *Functions from F are called attributes, and the pair U=(A,F) is called an information system.*

**Definition** **16.** *A problem over U is any (n+1) tuple $z=(\nu ,f_{1},\ldots ,f_{n})$, where n∈N∖{0}, $\nu :E_{k}^{n}\to P\left(N\right)$, and $f_{1},\ldots ,f_{n}\in F$.*

The problem *z* can be interpreted as a problem of searching for at least one number from the set $z\left(a\right)=\nu (f_{1}\left(a\right),\ldots ,f_{n}\left(a\right))$ for a given a∈A. We denote as Probl(U) the set of problems over the information system *U*.

We correspond to the problem *z* a decision table $T\left(z\right)\in M_{k}\left(F\right)$. This table has *n* columns labeled with attributes $f_{1},\ldots ,f_{n}$. A tuple $\bar{\delta }=\left(\delta_{1},\ldots ,\delta_{n}\right)\in E_{k}^{n}$ is a row of the table T(z) if and only if the system of equations

$$\left\{f_{1}\left(x\right)=\delta_{1},\ldots ,f_{n}\left(x\right)=\delta_{n}\right\}$$

has a solution from the set *A*. This row is labeled with the set of decisions $\nu (\bar{\delta })$. Let Tab(U)={T(z):z∈Probl(U)}. One can show that the set Tab(U) is a closed class of decision tables.

Closed classes of decision tables based on information systems are the most natural examples of closed classes. However, the notion of a closed class is essentially wider. In particular, the union $Tab\left(U_{1}\right)\cup Tab\left(U_{2}\right)$, where $U_{1}$ and $U_{2}$ are information systems, is a closed class, but generally, we cannot find an information system *U* such that $Tab\left(U\right)=Tab\left(U_{1}\right)\cup Tab\left(U_{2}\right)$.

### 2.5. Types of T Pairs

First, we define the notion of a t pair.

**Definition** **17.** *A pair (C,ψ), where C is a closed class of decision tables from $M_{k}\left(F\right)$, and ψ is a complexity measure over $M_{k}\left(F\right)$, is called a test pair (or t pair for short). If ψ is a limited complexity measure, then t pair (C,ψ) will be called a limited t pair.*

Let (C,ψ) be a t pair. We have three parameters $\psi^{i}\left(T\right),\psi^{d}\left(T\right)$, and $\psi^{a}\left(T\right)$ for any decision table T∈C. We now define functions that describe the relationships among these parameters. Let b,c∈{i,d,a}.

**Definition** **18.** *We define the partial functions $U_{C\psi }^{bc}:N\to N$ and $L_{C\psi }^{bc}:N\to N$ as*

$$\begin{array}{cc} & U_{C\psi }^{bc}\left(n\right)=\max\left\{\psi^{b}\left(T\right):T\in C,\psi^{c}\left(T\right)\leq n\right\},\\ & L_{C\psi }^{bc}\left(n\right)=\min\left\{\psi^{b}\left(T\right):T\in C,\psi^{c}\left(T\right)\geq n\right\}. \end{array}$$

If the value $U_{C\psi }^{bc}\left(n\right)$ is definite, then it is the unimprovable upper bound on the values $\psi^{b}\left(T\right)$ for tables T∈C satisfying $\psi^{c}\left(T\right)\leq n$. If the value $L_{C\psi }^{bc}\left(n\right)$ is definite, then it is the unimprovable lower bound on the values $\psi^{b}\left(T\right)$ for tables T∈C satisfying $\psi^{c}\left(T\right)\geq n$.

Let *g* be a partial function from N to N. We denote as Dom(g) the domain of *g*. Denote ${\mathrm{Dom}}^{+}\left(g\right)=\left\{n:n\in \mathrm{Dom}\left(g\right),g\left(n\right)\geq n\right\}$ and ${\mathrm{Dom}}^{-}\left(g\right)=\{n:n\in$Dom(g),g(n)≤n}.

**Definition** **19.** *Now, we define the value typ(g)∈{α,β,γ,δ,ϵ} as the type of g. Then, we have the following:*

- *If Dom(g) is an infinite set and g is bounded from the above function, then typ(g)=α.*
- *If Dom(g) is an infinite set, ${\mathrm{Dom}}^{+}\left(g\right)$ is a finite set, and g is unbounded from the above function, then typ(g)=β.*
- *If both sets ${\mathrm{Dom}}^{+}\left(g\right)$ and ${\mathrm{Dom}}^{-}\left(g\right)$ are infinite, then typ(g)=γ.*
- *If Dom(g) is an infinite set and ${\mathrm{Dom}}^{-}\left(g\right)$ is a finite set, then typ(g)=δ.*
- *If Dom(g) is a finite set, then typ(g)=ϵ.*

**Example** **8.** *One can show that typ(1)=α, $\mathrm{typ}(\left\lceil \log_{2}n\right\rceil )=\beta$, typ(n)=γ, $\mathrm{typ}(n^{2})=\delta$, and $\mathrm{typ}(\frac{1}{\left\lfloor 1/n\right\rfloor })=\epsilon$.*

**Definition** **20.** *We now define the table typ(C,ψ), which is called the type of t pair (C,ψ). This is a table with three rows and three columns, in which the rows from top to bottom and the columns from left to right are labeled with the indices i,d,a. The pair $\mathrm{typ}\left(L_{C\psi }^{bc}\right)\mathrm{typ}\left(U_{C\psi }^{bc}\right)$ is in the intersection of the row with index b∈{i,d,a} and the column with index c∈{i,d,a}.*

## 3. Main Results

The main problem investigated in this paper is finding all the types of t pairs and limited t pairs. The solution to this problem describes all the possible (in terms of functions $U_{C\psi }^{bc},L_{C\psi }^{bc}$ and types, b,c∈{i,d,a}) relationships among the complexity of decision tables, the minimum complexity of the nondeterministic decision trees for them, and the minimum complexity of the deterministic decision trees for these tables. We now define seven tables:

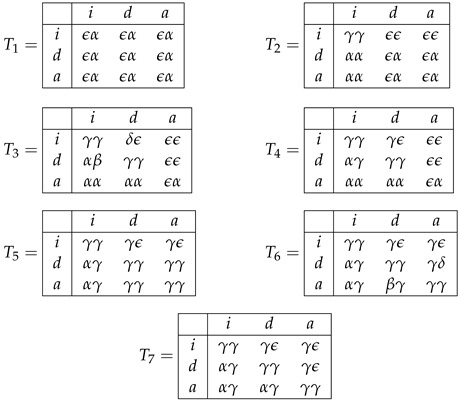

**Theorem** **1.** *For any t pair (C,ψ), the relation $\mathrm{typ}\left(C,\psi \right)\in \{T_{1},T_{2},T_{3},T_{4},T_{5},T_{6},T_{7}\}$ holds. For any i∈{1,2,3,4,5,6,7}, there exists a t pair (C,ψ) such that $\mathrm{typ}\left(C,\psi \right)=T_{i}$.*

**Theorem** **2.** *For any limited t pair (C,ψ), the relation $\mathrm{typ}\left(C,\psi \right)\in \{T_{2},T_{3},T_{5},T_{6},T_{7}\}$ holds. For any i∈{2,3,5,6,7}, there exists a limited t pair (C,h) such that $\mathrm{typ}\left(C,h\right)=T_{i}$.*

## 4. Possible Upper Types of T Pairs

We begin our study by considering the upper type of t pair, which is a simpler object than the type of t pair.

**Definition** **21.** *Let (C,ψ) be a t pair. We now define table ${\mathrm{typ}}_{u}\left(C,\psi \right)$, which will be called the upper type of t pair (C,ψ). This is a table with three rows and three columns, in which the rows from top to bottom and the columns from left to right are labeled with the indices i,d,a. The value $\mathrm{typ}(U_{C\psi }^{bc})$ is in the intersection of the row with index b∈{i,d,a} and the column with index c∈{i,d,a}. The table ${\mathrm{typ}}_{u}\left(C,\psi \right)$ is called the upper type of t pair (C,ψ).*

In this section, all possible upper types of t pairs are enumerated. We now define seven tables:

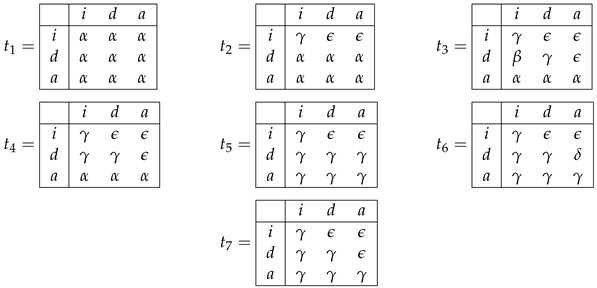

**Proposition** **1.** *For any t pair (C,ψ), the relation ${\mathrm{typ}}_{u}\left(C,\psi \right)\in \{t_{1},t_{2},t_{3},t_{4},t_{5},t_{6},t_{7}\}$ holds.*

**Proposition** **2.** *For any limited t pair (C,ψ), the relation ${\mathrm{typ}}_{u}\left(C,\psi \right)\in \{t_{2},t_{3},t_{5},t_{6},t_{7}\}$ holds.*

We divide the proofs of the propositions into a sequence of lemmas.

**Lemma** **1.** *Let T be a decision table from a set of decision tables $M_{k}\left(F\right)$, and let ψ be a complexity measure over $M_{k}\left(F\right)$. Then, the inequalities $\psi^{a}\left(T\right)\leq \psi^{d}\left(T\right)\leq \psi^{i}\left(T\right)$ hold.*

**Proof.**  Let the columns of table *T* be labeled with the attributes $f_{1},\ldots ,f_{n}$. It is not difficult to construct a deterministic decision tree $\Gamma_{0}$ for table *T*, which sequentially computes the values of attributes $f_{1},\ldots ,f_{n}$. Evidently, $\psi \left(\Gamma_{0}\right)=\psi^{i}\left(T\right)$. Therefore, $\psi^{d}\left(T\right)\leq \psi^{i}\left(T\right)$. If a decision tree Γ is a deterministic decision tree for *T*, then Γ is a nondeterministic decision tree for *T*. Therefore, $\psi^{a}\left(T\right)\leq \psi^{d}\left(T\right)$. □

Let (C,ψ) be a t pair, n∈N, and b,c∈{i,d,a}. The notation $U_{C\psi }^{bc}\left(n\right)=\infty$ means that the set $X=\{\psi^{b}\left(T\right):T\in C,\psi^{c}\left(T\right)\leq n\}$ is infinite. The notation $U_{C\psi }^{bc}\left(n\right)=⌀$ means that the set *X* is empty. Evidently, if $U_{C\psi }^{bc}\left(n\right)=\infty$, then $U_{C\psi }^{bc}\left(n+1\right)=\infty$. It is not difficult to prove the following statement.

**Lemma** **2.** *Let (C,ψ) be a t pair, and b,c∈{i,d,a}. Then, we have the following:*

*(a) If there exists n∈N such that $U_{C\psi }^{bc}\left(n\right)=\infty$, then $\mathrm{typ}(U_{C\psi }^{bc})=\epsilon$.*

*(b) If there is no n∈N such that $U_{C\psi }^{bc}\left(n\right)=\infty$, then $\mathrm{Dom}\left(U_{C\psi }^{bc}\right)=\{n:n\in$$\left.N,n\geq n_{0}\right\}$, where $n_{0}=\min\left\{\psi^{c}\left(T\right):T\in C\right\}$.*

Let (C,ψ) be a t pair, and b,c,e,f∈{i,d,a}. The notation $U_{C\psi }^{bc}◃U_{C\psi }^{ef}$ means that, for any n∈N, the following statements hold:

(a) If the value $U_{C\psi }^{bc}\left(n\right)$ is definite, then either $U_{C\psi }^{ef}\left(n\right)=\infty$ or the value $U_{C\psi }^{ef}\left(n\right)$ is definite, and the inequality $U_{C\psi }^{bc}\left(n\right)\leq U_{C\psi }^{ef}\left(n\right)$ holds.

(b) If $U_{C\psi }^{bc}\left(n\right)=\infty$, then $U_{C\psi }^{ef}\left(n\right)=\infty$.

Let ⪯ be a linear order on the set {α,β,γ,δ,ϵ} such that α⪯β⪯γ⪯δ⪯ϵ.

**Lemma** **3.** *Let (C,ψ) be a t pair. Then, $\mathrm{typ}\left(U_{C\psi }^{bi}\right)⪯\mathrm{typ}\left(U_{C\psi }^{bd}\right)⪯\mathrm{typ}\left(U_{C\psi }^{ba}\right)$ and $\mathrm{typ}\left(U_{C\psi }^{ab}\right)⪯\mathrm{typ}\left(U_{C\psi }^{db}\right)⪯\mathrm{typ}\left(U_{C\psi }^{ib}\right)$ for any b∈{i,d,a}.*

**Proof.**  From the definition of the functions $U_{C\psi }^{bc},b,c\in \left\{i,d,a\right\}$ and from Lemma 1, it follows that $U_{C\psi }^{bi}◃U_{C\psi }^{bd}◃U_{C\psi }^{ba}$ and $U_{C\psi }^{ab}◃U_{C\psi }^{db}◃U_{C\psi }^{ib}$ for any b∈{i,d,a}. Using these relations and Lemma 2, we obtain the statement of the lemma. □

**Lemma** **4.** *Let (C,ψ) be a t pair, and b,c∈{i,d,a}. Then, we have the following:*

*(a) $\mathrm{typ}(U_{C\psi }^{bc})=\alpha$ if and only if the function $\psi^{b}$ is bounded from above on the closed class C.*

*(b) If the function $\psi^{b}$ is unbounded from above on C, then $\mathrm{typ}(U_{C\psi }^{bb})=\gamma$.*

**Proof.**  The statement (a) is obvious. For (b), let the function $\psi^{b}$ be unbounded from above on C. One can show that in this case the equality $U_{C\psi }^{bb}\left(n\right)=n$ holds for infinitely many n∈N. Therefore, $\mathrm{typ}(U_{C\psi }^{bb})=\gamma$. □

**Corollary** **1.** *Let (C,ψ) be a t pair, and b∈{i,d,a}. Then, $\mathrm{typ}\left(U_{C\psi }^{bb}\right)\in \left\{\alpha ,\gamma \right\}$.*

**Lemma** **5.** *Let (C,ψ) be a t pair, and $\mathrm{typ}(U_{C\psi }^{ii})\neq \alpha$. Then,*

$$\mathrm{typ}\left(U_{C\psi }^{id}\right)=\mathrm{typ}\left(U_{C\psi }^{ia}\right)=\epsilon .$$

**Proof.**  Using Lemma 4, we conclude that the function $\psi^{i}$ is unbounded from above on C. Let m∈N. Then, there exists a decision table T∈C for which the inequality $\psi^{i}\left(T\right)\geq m$ holds. Let us consider a degenerate decision table $T^{\prime }\in C$ obtained from *T* by replacing the sets of decisions attached to the rows by the set {0}. It is clear that $\psi^{i}\left(T^{\prime }\right)\geq m$. Let Γ be a decision tree that consists of the root, the terminal node labeled with 0, and the edge connecting these two nodes. One can show that Γ is a deterministic decision tree for the table $T^{\prime }$. Therefore, $\psi^{a}\left(T^{\prime }\right)\leq \psi^{d}\left(T^{\prime }\right)\leq \psi \left(\Gamma \right)=\psi \left(\lambda \right)$. Taking into account that *m* is an arbitrary number from N, we obtain $U_{C\psi }^{id}\left(\psi \left(\lambda \right)\right)=\infty$ and $U_{C\psi }^{ia}\left(\psi \left(\lambda \right)\right)=\infty$. Using Lemma 2, we conclude that $\mathrm{typ}\left(U_{C\psi }^{id}\right)=\mathrm{typ}\left(U_{C\psi }^{ia}\right)=\epsilon$. □

**Example** **9.** *Let us consider a t pair $\left(C_{0},h\right)$, where $C_{0}$ is a closed class described in Example 5. It is clear that the function $h^{i}$ is unbounded from above on $C_{0}$, and the functions $h^{a}$ and $h^{d}$ are bounded from above on $C_{0}$. Using Lemma 4, we obtain that $\mathrm{typ}\left(U_{C_{0}h}^{ab}\right)=\mathrm{typ}\left(U_{C_{0}h}^{db}\right)=\alpha$ for any b∈{i,d,a}, and $\mathrm{typ}(U_{C_{0}h}^{ii})=\gamma$. Using Lemma 5, $\mathrm{typ}\left(U_{C_{0}h}^{id}\right)=\mathrm{typ}\left(U_{C_{0}h}^{ia}\right)=\epsilon$. Therefore, ${\mathrm{typ}}_{u}\left(C_{0},h\right)=t_{2}$.*

**Lemma** **6.** *Let (C,ψ) be a t pair. Then, $\mathrm{typ}\left(U_{C\psi }^{ai}\right)\in \left\{\alpha ,\gamma \right\}$.*

**Proof.**  Using Lemma 3 and Corollary 1, we obtain $\mathrm{typ}\left(U_{C\psi }^{ai}\right)\in \left\{\alpha ,\beta ,\gamma \right\}$. Using Lemma 2, $\mathrm{Dom}\left(U_{C\psi }^{ai}\right)=\left\{n:n\in N,n\geq n_{0}\right\}$ for some $n_{0}\in N$. Set $D=\mathrm{Dom}(U_{C\psi }^{ai})$. Assume that $\mathrm{typ}(U_{C\psi }^{ai})=\beta$. Then, there exists m∈D such that $U_{C\psi }^{ai}\left(n\right)<n$ for any n∈D,n>m. Let us prove by induction on *n* that, for any decision table *T* from C, if $\psi^{i}\left(T\right)\leq n$, then $\psi^{a}\left(T\right)\leq m_{0}$, where $m_{0}=\max\left\{m,\psi \left(\lambda \right)\right\}$. Using Lemma 1, we conclude that the considered statement holds under the condition n≤m. Let it hold for some n,n≥m. Let us show that this statement holds for n+1 too. Let T∈C, $\psi^{i}\left(T\right)\leq n+1$, and let the columns of the table *T* be labeled with the attributes $f_{i_{1}},\ldots ,f_{i_{k}}$. Since n+1>m, we obtain $\psi^{a}\left(T\right)\leq n$. Let Γ be a nondeterministic decision tree for the table *T*, and $\psi \left(\Gamma \right)=\psi^{a}\left(T\right)$. Assume that in Γ, there exists a complete path ξ in which there are no worker nodes. In this case, a decision tree that consists of the root, the terminal node labeled with τ(ξ), and the edge connecting these two nodes is a nondeterministic decision tree for the table *T*. Therefore, $\psi^{a}\left(T\right)\leq \psi \left(\lambda \right)\leq m_{0}$. Assume now that each complete path in the decision tree Γ contains a worker node. Let ξ∈Path(Γ),Δ(Tπ(ξ))≠⌀, $\xi =v_{0},d_{0},\ldots ,v_{p},d_{p},v_{p+1}$ and, for i=1,…,p, the node $v_{i}$ is labeled with the attribute $f_{i}$, and the edge $d_{i}$ is labeled with the number $\delta_{i}$. Let the decision table $T^{\prime }$ be obtained from the decision table *T* using the operations of the permutation of columns and the duplication of columns so that its columns are labeled with attributes $f_{1},\ldots ,f_{p},f_{i_{1}},\ldots ,f_{i_{k}}$. We obtain the decision table $T^{\prime \prime }$ from $T^{\prime }$ by removal of the last *k* columns. Let us denote as $T_{\xi }$ the decision table obtained from $T^{\prime \prime }$ by changing the set of decisions corresponding to the row $\left(\delta_{1},\ldots ,\delta_{p}\right)$ with {τ(ξ)} and for the remaining rows with {τ(ξ)+1}. It is clear that $\psi^{i}\left(T_{\xi }\right)\leq n$. Using the inductive hypothesis, we conclude that there exists a nondeterministic decision tree $\Gamma_{\xi }$ for the table $T_{\xi }$ such that $\psi \left(\Gamma_{\xi }\right)\leq m_{0}$. We denote as ${\tilde{\Gamma }}_{\xi }$ a tree obtained from $\Gamma_{\xi }$ by the removal of all the nodes and edges that satisfy the following condition: there is not a complete path $\xi^{\prime }$ in $\Gamma_{\xi }$ that contains this node or edge and for which $\tau \left(\xi^{\prime }\right)=\tau \left(\xi \right)$. Let $\left\{\xi :\xi \in \mathrm{Path}\left(\Gamma \right),\Delta \left(T\pi \left(\xi \right)\right)\neq ⌀\right\}=\left\{\xi_{1},\ldots ,\xi_{r}\right\}$. Let us identify the roots of the trees ${\tilde{\Gamma }}_{\xi_{1}},\ldots ,{\tilde{\Gamma }}_{\xi_{r}}$. We denote as *G* the obtained tree. It is not difficult to show that *G* is a nondeterministic decision tree for the table *T*, and $\psi \left(G\right)\leq m_{0}$. Thus, the considered statement holds. Using Lemma 4, we conclude that $\mathrm{typ}(U_{C\psi }^{ai})=\alpha$. The obtained contradiction shows that $\mathrm{typ}\left(U_{C\psi }^{ai}\right)\in \left\{\alpha ,\gamma \right\}$. □

Let *T* be a decision table from $M_{k}\left(F\right)$. We now give the definitions of the parameters N(T) and M(T) of the table *T*.

**Definition** **22.** *We denote as N(T) the number of rows in the table T.*

**Definition** **23.** *Let the columns of table T be labeled with the attributes $f_{1},\ldots ,f_{n}\in F$. We now define the parameter M(T). If table T is degenerate, then M(T)=0. Let T now be a nondegenerate table, and $\bar{\delta }=\left(\delta_{1},\ldots ,\delta_{n}\right)\in E_{k}^{n}$. Then, $M(T,\bar{\delta })$ is the minimum natural m such that there exist attributes $f_{i_{1}},\ldots ,f_{i_{m}}\in At\left(T\right)$ for which $T\left(f_{i_{1}},\delta_{i_{1}}\right)\cdots \left(f_{i_{m}},\delta_{i_{m}}\right)$ is a degenerate table. We denote $M\left(T\right)=\max\{M\left(T,\bar{\delta }\right):\bar{\delta }\in E_{k}^{n}\}$.*

The following statement follows immediately from Theorem 3.5 [23].

**Lemma** **7.** *Let T be a nonempty decision table from $M_{k}\left(F\right)$ in which each row is labeled with a set containing only one decision. Then,*

$$h^{d}\left(T\right)\leq M\left(T\right)\log_{2}N\left(T\right).$$

**Lemma** **8.** *Let (C,ψ) be a limited t pair, and $\mathrm{typ}(U_{C\psi }^{ai})=\alpha$. Then, $\mathrm{typ}(U_{C\psi }^{di})\in${α,β}.*

**Proof.**  Using Lemma 4, we conclude that there exists r∈N such that the inequality $\psi^{a}\left(T\right)\leq r$ holds for any table T∈C. □

Let *T* be a nonempty table from C in which the columns are labeled with the attributes $f_{1},\ldots ,f_{n}$ and $\bar{\delta }=\left(\delta_{1},\ldots ,\delta_{n}\right)\in E_{k}^{n}$. We now show that there exist attributes $f_{i_{1}},\ldots ,f_{i_{m}}\in At\left(T\right)$ such that the subtable $T\left(\bar{\delta }\right)=T\left(f_{1},\delta_{1}\right)\cdots \left(f_{n},\delta_{n}\right)$ is equal to the subtable $T\left(f_{i_{1}},\delta_{i_{1}}\right)\cdots \left(f_{i_{m}},\delta_{i_{m}}\right)$, and m≤r if $\bar{\delta }$ is a row of *T*; as well, m≤r+1 if $\bar{\delta }$ is not a row of *T*.

Let $\bar{\delta }$ be a row of *T*. Let us change the set of decisions attached to the row $\bar{\delta }$ with the set {1} and for the remaining rows of *T* with the set {0}. We denote the obtained table as $T^{\prime }$. It is clear that $T^{\prime }\in C$. Taking into account that $\psi^{a}\left(T^{\prime }\right)\leq r$ and the complexity measure ψ has the property (c), it is not difficult to show that there exist attributes $f_{i_{1}},\ldots ,f_{i_{m}}\in At\left(T^{\prime }\right)=At\left(T\right)$ such that m≤r, and $T^{\prime }\left(f_{i_{1}},\delta_{i_{1}}\right)\cdots \left(f_{i_{m}},\delta_{i_{m}}\right)$ contains only the row $\bar{\delta }$. From here, it follows that $T\left(\bar{\delta }\right)=T\left(f_{i_{1}},\delta_{i_{1}}\right)\cdots \left(f_{i_{m}},\delta_{i_{m}}\right)$.

Let $\bar{\delta }$ be not a row of *T*. Let us show that there exist attributes $f_{i_{1}},\ldots ,f_{i_{m}}\in At\left(T\right)$ such that m≤r+1, and the subtable $T\left(f_{i_{1}},\delta_{i_{1}}\right)\cdots \left(f_{i_{m}},\delta_{i_{m}}\right)$ is empty. If $T(f_{1},\delta_{1})$ is empty, then the considered statement holds. Otherwise, there exists q∈{1,…,n−1} such that the subtable $T\left(f_{1},\delta_{1}\right)\cdots \left(f_{q},\delta_{q}\right)$ is nonempty, but the subtable $T\left(f_{1},\delta_{1}\right)\cdots \left(f_{q+1},\delta_{q+1}\right)$ is empty. We denote as $T^{\prime }$ the table obtained from *T* by the removal of the attributes $f_{q+1},\ldots ,f_{n}$. It is clear that $T^{\prime }\in C$, and $\left(\delta_{1},\ldots ,\delta_{q}\right)$ is a row of $T^{\prime }$. According to what has been proven above, there exist attributes $f_{i_{1}},\ldots ,f_{i_{p}}\in \left\{f_{1},\ldots ,f_{q}\right\}$ such that

$$T^{\prime }\left(f_{i_{1}},\delta_{i_{1}}\right)\cdots \left(f_{i_{p}},\delta_{i_{p}}\right)=T^{\prime }\left(f_{1},\delta_{1}\right)\cdots \left(f_{q},\delta_{q}\right)$$

and p≤r. Using this fact, one can show that $T\left(f_{i_{1}},\delta_{i_{1}}\right)\cdots \left(f_{i_{p}},\delta_{i_{p}}\right)\left(f_{q+1},\delta_{q+1}\right)$ is empty and is equal to $T(\bar{\delta })$.

Let $T_{1}\in C$. We denote as $T_{2}$ the decision table obtained from $T_{1}$ by the removal of all the columns in which all the numbers are equal. Let the columns of $T_{2}$ be labeled with attributes $f_{1},\ldots ,f_{n}$. We now consider the decision table $T_{3}$, which is obtained from $T_{2}$ by changing the decisions so that the decision set attached to each row of table $T_{3}$ contains only one decision and, for any two non-equal rows, the corresponding decisions are different. It is clear that $T_{3}\in C$. It is not difficult to show that $\psi^{d}\left(T_{1}\right)\leq \psi^{d}\left(T_{2}\right)\leq \psi^{d}\left(T_{3}\right)$.

We now show that the inequality ψ(f)≤r holds for any attribute $f\in At(T_{3})$. Let us denote as $T^{\prime }$ the decision table obtained from $T_{3}$ by the removal of all the columns except the column labeled with the attribute *f*. If there is more than one column in $T_{3}$, which is labeled with the attribute *f*, then we keep only one of them. Let the decision table $T_{f}$ be obtained from $T^{\prime }$ by changing the set of decisions for each row (δ) with the set of decisions {δ}. It is clear that $T_{f}\in C$. Let Γ be a nondeterministic decision tree for the table $T_{f}$, and $\psi \left(\Gamma \right)=\psi^{a}\left(T_{f}\right)\leq r$. Since the column *f* contains different numbers, we have f∈At(Γ). Using the property (b) of the complexity measure ψ, we obtain ψ(Γ)≥ψ(f). Consequently, ψ(f)≤r.

Taking into account that, for any $\bar{\delta }\in \Delta \left(T_{3}\right)$, there exist attributes $f_{i_{1}},\ldots ,$$f_{i_{m}}\in \left\{f_{1},\ldots ,f_{n}\right\}$ such that m≤r, and $T_{3}\left(f_{i_{1}},\delta_{i_{1}}\right)\cdots \left(f_{i_{m}},\delta_{i_{m}}\right)$ contains only the row $\bar{\delta }$, it is not difficult to show that

(1) $$N\left(T_{3}\right)\leq n^{r}\cdot k^{r}.$$

According to what has been proven above, for any $\bar{\delta }\in E_{k}^{n}$, there exist attributes $f_{i_{1}},\ldots ,$$f_{i_{m}}\in \left\{f_{1},\ldots ,f_{n}\right\}$ such that m≤r+1, and $T_{3}\left(f_{i_{1}},\delta_{i_{1}}\right)\cdots \left(f_{i_{m}},\delta_{i_{m}}\right)=T_{3}\left(f_{1},\delta_{1}\right)$$\cdots (f_{n},\delta_{n})$. Taking into account this equality, one can show that

(2) $$M(T_{3})\leq r+1.$$

Using Lemma 7, as well as inequalities (1) and (2), we conclude that there exists a deterministic decision tree Γ for the table $T_{3}$ with $h\left(\Gamma \right)\leq M\left(T_{3}\right)\log_{2}N\left(T_{3}\right)\leq {\left(r+1\right)}^{2}\log_{2}\left(kn\right)$. Taking into account that ψ(f)≤r for any attribute $f\in At(T_{3})$ and that the complexity measure ψ has the property (a), we obtain

$$\psi^{d}\left(T_{3}\right)\leq {\left(r+1\right)}^{3}\log_{2}\left(kn\right).$$

Consequently, $\psi^{d}\left(T_{1}\right)\leq {\left(r+1\right)}^{3}\log_{2}\left(kn\right)$. Taking into account that the complexity measure ψ has the property (c), we obtain $\psi^{i}\left(T_{1}\right)\geq n$. Since $T_{1}$ is an arbitrary decision table from C, we have that ${\mathrm{Dom}}^{+}\left(U_{C\psi }^{di}\right)$ is a finite set. Therefore, $\mathrm{typ}(U_{U\psi }^{di})\neq \gamma$. Using Lemma 3 and Corollary 1, we obtain $\mathrm{typ}\left(U_{C\psi }^{di}\right)\in \left\{\alpha ,\beta \right\}$. □

**Proof of Proposition** **1.** Let (C,ψ) be a t pair. Using Corollary 1, we conclude that $\mathrm{typ}\left(U_{C\psi }^{ii}\right)\in \left\{\alpha ,\gamma \right\}$. Using Corollary 1 and Lemma 3, we obtain $\mathrm{typ}\left(U_{C\psi }^{di}\right)\in \left\{\alpha ,\beta ,\gamma \right\}$. From Lemma 6, it follows that $\mathrm{typ}\left(U_{C\psi }^{ai}\right)\in \left\{\alpha ,\gamma \right\}$. Then, we have the following:

(a) Let $\mathrm{typ}(U_{C\psi }^{ii})=\alpha$. Using Lemmas 3 and 4, we obtain ${\mathrm{typ}}_{u}\left(C,\psi \right)=t_{1}$.

(b) Let $\mathrm{typ}(U_{C\psi }^{ii})=\gamma$ and $\mathrm{typ}(U_{C\psi }^{di})=\alpha$. Using Lemmas 3, 4, and 5, we obtain ${\mathrm{typ}}_{u}\left(C,\psi \right)=t_{2}$.

(c) Let $\mathrm{typ}(U_{C\psi }^{ii})=\gamma$ and $\mathrm{typ}(U_{C\psi }^{di})=\beta$. From Lemma 5, it follows that $\mathrm{typ}\left(U_{C\psi }^{id}\right)=\mathrm{typ}\left(U_{C\psi }^{ia}\right)=\epsilon$. Using Lemmas 3 and 6, we obtain $\mathrm{typ}(U_{C\psi }^{ai})=\alpha$. From this equality and from Lemma 4, it follows that $\mathrm{typ}\left(U_{C\psi }^{ad}\right)=\mathrm{typ}\left(U_{C\psi }^{aa}\right)=$α. Using the equality $\mathrm{typ}(U_{C\psi }^{di})=\beta$, Lemma 3, and Corollary 1, we obtain $\mathrm{typ}(U_{C\psi }^{dd})=\gamma$. From the equalities, $\mathrm{typ}\left(U_{C\psi }^{dd}\right)=\gamma ,\mathrm{typ}\left(U_{C\psi }^{aa}\right)=\alpha$ and from Lemmas 2 and 4, it follows that $\mathrm{typ}(U_{C\psi }^{da})=\epsilon$. Thus, ${\mathrm{typ}}_{u}\left(C,\psi \right)=t_{3}$.

(d) Let $\mathrm{typ}\left(U_{C\psi }^{ii}\right)=\mathrm{typ}\left(U_{C\psi }^{di}\right)=\gamma$ and $\mathrm{typ}(U_{C\psi }^{ai})=\alpha$. Using Lemma 5, we obtain $\mathrm{typ}\left(U_{C\psi }^{id}\right)=\mathrm{typ}\left(U_{C\psi }^{ia}\right)=\epsilon$. From Lemma 4, it follows that $\mathrm{typ}(U_{C\psi }^{ad})=$$\mathrm{typ}(U_{C\psi }^{aa})=\alpha$. Using Lemma 3 and Corollary 1, we obtain $\mathrm{typ}(U_{C\psi }^{dd})=\gamma$. From this equality, equality $\mathrm{typ}(U_{C\psi }^{aa})=\alpha$, and from Lemmas 2 and 4, it follows that $\mathrm{typ}(U_{C\psi }^{da})=\epsilon$. Thus, ${\mathrm{typ}}_{u}\left(C,\psi \right)=t_{4}$.

(e) Let $\mathrm{typ}\left(U_{C\psi }^{ii}\right)=\mathrm{typ}\left(U_{C\psi }^{di}\right)=\mathrm{typ}\left(U_{C\psi }^{ai}\right)=\gamma$. Using Lemma 5, we conclude that $\mathrm{typ}\left(U_{C\psi }^{id}\right)=\mathrm{typ}\left(U_{C\psi }^{ia}\right)=\epsilon$. Using Lemma 3 and Corollary 1, we obtain $\mathrm{typ}\left(U_{C\psi }^{dd}\right)=\mathrm{typ}\left(U_{C\psi }^{ad}\right)=\mathrm{typ}\left(U_{C\psi }^{aa}\right)=\gamma$. Using Lemma 3, we obtain $\mathrm{typ}(U_{C\psi }^{da})\in${γ,δ,ϵ}. Therefore, ${\mathrm{typ}}_{u}\left(C,\psi \right)\in \left\{t_{5},t_{6},t_{7}\right\}$. □

**Proof of Proposition** **2.** Let (C,ψ) be a limited t pair. Taking into account that the complexity measure ψ has the property (c) and using Lemma 4, we obtain $\mathrm{typ}(U_{C\psi }^{ii})\neq \alpha$. Therefore, ${\mathrm{typ}}_{u}\left(C,\psi \right)\neq t_{1}$. Using Lemma 8, we obtain ${\mathrm{typ}}_{u}\left(C,\psi \right)\neq t_{4}$. From these relations and Proposition 1, it follows that the statement of the proposition holds. □

## 5. Realizable Upper Types of T Pairs

In this section, all realizable upper types of t pairs are enumerated.

**Proposition** **3.** *For any i∈{1,2,3,4,5,6,7}, there exists a t pair (C,ψ) such that*

$${\mathrm{typ}}_{u}\left(C,\psi \right)=t_{i}.$$

**Proposition** **4.** *For any i∈{2,3,5,6,7}, there exists a limited t pair (C,h) such that*

$${\mathrm{typ}}_{u}\left(C,h\right)=t_{i}.$$

The proofs of these propositions are based on the results obtained for information systems [22].

Let U=(A,F) be an information system, where the attributes from *F* have values from $E_{k}$, and ψ is a complexity measure over *U* [22]. Note that ψ is also a complexity measure over the set of decision tables $M_{k}\left(F\right)$. Let $z=(\nu ,f_{1},\ldots ,f_{n})$ be a problem over *U*. In [22], three parameters of the problem *z* were defined: $\psi_{U}^{i}\left(z\right)=\psi \left(f_{1}\cdots f_{n}\right)$ was called the complexity of the problem *z* description, $\psi_{U}^{d}\left(z\right)$ was called the minimum complexity of a decision tree with attributes from the set $\left\{f_{1},\ldots ,f_{n}\right\}$—which solves the problem *z* deterministically—and $\psi_{U}^{a}\left(z\right)$ was called the minimum complexity of a decision tree with attributes from the set $\left\{f_{1},\ldots ,f_{n}\right\}$, which solves the problem *z* nondeterministically.

Let b,c∈{i,d,a}. In [22], the partial function $U_{U\psi }^{bc}:N\to N$ was defined as follows:

$$U_{U\psi }^{bc}\left(n\right)=\max\left\{\psi_{U}^{b}\left(z\right):z\in Probl\left(U\right),\psi_{U}^{c}\left(z\right)\leq n\right\}.$$

The table ${\mathrm{typ}}_{lu}\left(U,\psi \right)$ for the pair (U,ψ) was defined in [22] as follows: this is a table with three rows and three columns, in which the rows from top to bottom and the columns from left to right are labeled with the indices i,d,a. The value $\mathrm{typ}(U_{U\psi }^{bc})$ is in the intersection of the row with the index b∈{i,d,a} and the column with the index c∈{i,d,a}.

We now prove the following proposition:

**Proposition** **5.** *Let U be an information system and ψ be a complexity measure over U. Then,*

$${\mathrm{typ}}_{lu}\left(U,\psi \right)={\mathrm{typ}}_{u}\left(Tab\left(U\right),\psi \right).$$

**Proof.**  Let $z=(\nu ,f_{1},\ldots ,f_{n})$ be a problem over *U* and T(z) be the decision table corresponding to this problem. It is easy to see that $\psi_{U}^{i}\left(z\right)=\psi^{i}\left(T\left(z\right)\right)$. One can show that the set of decision trees solving the problem *z* nondeterministically and using only the attributes from the set $\left\{f_{1},\ldots ,f_{n}\right\}$ (see corresponding definitions in [22]) is equal to the set of nondeterministic decision trees for the table T(z). From here, it follows that $\psi_{U}^{a}\left(z\right)=\psi^{a}\left(T\left(z\right)\right)$ and $\psi_{U}^{d}\left(z\right)=\psi^{d}\left(T\left(z\right)\right)$. Using these equalities, we can show that ${\mathrm{typ}}_{lu}\left(U,\psi \right)={\mathrm{typ}}_{u}\left(Tab\left(U\right),\psi \right)$. □

This proposition allows us to transfer the results obtained for information systems in [22] to the case of closed classes of decision tables. Before each of the following seven lemmas, we define a pair (U,ψ), where *U* is an information system, and ψ is a complexity measure over *U*.

Let us define a pair $\left(U_{1},\pi \right)$ as follows: $U_{1}=\left(N,F_{1}\right)$, where $F_{1}=\left\{f\right\}$, f≡0, and π≡0.

**Lemma** **9.** *${\mathrm{typ}}_{u}\left(Tab\left(U_{1}\right),\pi \right)=t_{1}$.*

**Proof.**  From Lemma 4.1 [22], it follows that ${\mathrm{typ}}_{lu}\left(U_{1},\pi \right)=t_{1}$. Using Proposition 5, we obtain ${\mathrm{typ}}_{u}\left(Tab\left(U_{1}\right),\pi \right)=t_{1}$. □

Let us define a pair $\left(U_{2},h\right)$ as follows: $U_{2}=\left(N,F_{2}\right)$, where $F_{2}=F_{1}$.

**Lemma** **10.** *${\mathrm{typ}}_{u}\left(Tab\left(U_{2}\right),h\right)=t_{2}$.*

**Proof.**  From Lemma 4.2 [22], it follows that ${\mathrm{typ}}_{lu}\left(U_{2},h\right)=t_{2}$. Using Proposition 5, we obtain ${\mathrm{typ}}_{u}\left(Tab\left(U_{2}\right),h\right)=t_{2}$. □

Let us define a pair $\left(U_{3},h\right)$ as follows: $U_{3}=\left(N,F_{3}\right)$, where $F_{3}=\left\{l_{i}:i\in N\setminus \left\{0\right\}\right\}$ and, for any i∈N∖{0},j∈N, if j≤i, then $l_{i}\left(j\right)=0$, and if j>i, then $l_{i}\left(j\right)=1$.

**Lemma** **11.** *${\mathrm{typ}}_{u}\left(Tab\left(U_{3}\right),h\right)=t_{3}$.*

**Proof.**  From Lemma 4.3 [22], it follows that ${\mathrm{typ}}_{lu}\left(U_{3},h\right)=t_{3}$. Using Proposition 5, we obtain ${\mathrm{typ}}_{u}\left(Tab\left(U_{3}\right),h\right)=t_{3}$. □

Let us define a pair $\left(U_{4},\mu \right)$ as follows: $U_{4}=\left(N,F_{4}\right)$, where $F_{4}=F_{3},\mu \left(\lambda \right)=$$0,\mu (l_{i_{1}}\cdots l_{i_{m}})=1$ if m=1 or m=2, and $i_{1}>i_{2},\mu \left(l_{i_{1}}\cdots l_{i_{m}}\right)=\max\left\{i_{1},\ldots ,i_{m}\right\}$ in other cases.

**Lemma** **12.** *${\mathrm{typ}}_{u}\left(Tab\left(U_{4}\right),\mu \right)=t_{4}$.*

**Proof.**  From Lemma 4.4 [22], it follows that ${\mathrm{typ}}_{lu}\left(U_{4},\mu \right)=t_{4}$. Using Proposition 5, we obtain ${\mathrm{typ}}_{u}\left(Tab\left(U_{4}\right),\mu \right)=t_{4}$. □

Let us define a pair $\left(U_{5},h\right)$ as follows: $U_{5}=\left(N,F_{5}\right)$, where $F_{5}=\{f_{i}:i\in$N∖{0}} and, for any i∈N∖{0},j∈N, if i=j, then $f_{i}\left(j\right)=1$, and if i≠j, then $f_{i}\left(j\right)=0$.

**Lemma** **13.** *${\mathrm{typ}}_{u}\left(Tab\left(U_{5}\right),h\right)=t_{5}$.*

**Proof.**  From Lemma 4.5 [22], it follows that ${\mathrm{typ}}_{lu}\left(U_{5},h\right)=t_{5}$. Using Proposition 5, we obtain ${\mathrm{typ}}_{u}\left(Tab\left(U_{5}\right),h\right)=t_{5}$. □

Let us define a pair $\left(U_{6},h\right)$ as follows: $U_{6}=\left(N,F_{6}\right)$, where $F_{6}=F_{5}\cup G$, $G=\{g_{2i+1}:i\in N\}$ and, for any i∈N,j∈N, if j∈{2i+1,2i+2}, then $g_{2i+1}\left(j\right)=1$, and if j∉{2i+1,2i+2}, then $g_{2i+1}\left(j\right)=0$.

**Lemma** **14.** *${\mathrm{typ}}_{u}\left(Tab\left(U_{6}\right),h\right)=t_{6}$.*

**Proof.**  From Lemma 4.6 [22], it follows that ${\mathrm{typ}}_{lu}\left(U_{6},h\right)=t_{6}$. Using Proposition 5, we obtain ${\mathrm{typ}}_{u}\left(Tab\left(U_{6}\right),h\right)=t_{6}$. □

Let us define a pair $\left(U_{7},h\right)$ as follows: $U_{7}=\left(N,F_{7}\right)$, where $F_{7}=F_{3}\cup F_{5}$.

**Lemma** **15.** *${\mathrm{typ}}_{u}\left(Tab\left(U_{7}\right),h\right)=t_{7}$.*

**Proof.**  From Lemma 4.7 [22], it follows that ${\mathrm{typ}}_{lu}\left(U_{7},h\right)=t_{7}$. Using Proposition 5, we obtain ${\mathrm{typ}}_{u}\left(Tab\left(U_{7}\right),h\right)=t_{7}$. □

**Proof of Proposition** **3.** The statement of the proposition follows from Lemmas 9–15. □

**Proof of Proposition** **4.** The statement of the proposition follows from Lemmas 10, 11, 13, 14, and 15. □

## 6. Union of T Pairs

In this section, we define a union of two t pairs, which is also a t pair, and study its upper type. Let $\tau_{1}=\left(C_{1},\psi_{1}\right)$ and $\tau_{2}=\left(C_{2},\psi_{2}\right)$ be t pairs, where $C_{1}\subseteq M_{k_{1}}\left(F_{1}\right)$, and $C_{2}\subseteq M_{k_{2}}\left(F_{2}\right)$. These two t pairs are called *compatible* if $F_{1}\cap F_{2}=⌀$ and $\psi_{1}\left(\lambda \right)=\psi_{2}\left(\lambda \right)$. We now define a t pair τ=(C,ψ), which is called a *union* of compatible t pairs $\tau_{1}$ and $\tau_{2}$.

**Definition** **24.** The closed class C in τ is defined as follows: $C=C_{1}\cup C_{2}\subseteq M_{\max(k_{1},k_{2})}\left(F_{1}\cup F_{2}\right)$. The complexity measure ψ in τ is defined for any word $\alpha \in {\left(F_{1}\cup F_{2}\right)}^{*}$ in the following way: if $\alpha \in F_{1}^{*}$, then $\psi \left(\alpha \right)=\psi_{1}\left(\alpha \right)$; if $\alpha \in F_{2}^{*}$, then $\psi \left(\alpha \right)=\psi_{2}\left(\alpha \right)$; if α contains letters from both $F_{1}$ and $F_{2}$, then ψ(α) can have an arbitrary value from N. In particular, if $\psi_{1}=\psi_{2}=h$, then with ψ we can use the depth h.

We now consider the upper type of t pair τ=(C,ψ). We denote as $\tilde{\max}$ the function maximum for the linear order α⪯β⪯γ⪯δ⪯ϵ.

**Theorem** **3.** *The equality $\mathrm{typ}\left(U_{C\psi }^{bc}\right)=\tilde{\max}\left(\mathrm{typ}\left(U_{C_{1}\psi_{1}}^{bc}\right),\mathrm{typ}\left(U_{C_{2}\psi_{2}}^{bc}\right)\right)$ holds for any b,c∈{i,d,a}, except for the case that bc=da and $\mathrm{typ}\left(U_{C_{1}\psi_{1}}^{da}\right)=\mathrm{typ}\left(U_{C_{2}\psi_{2}}^{da}\right)=\gamma$. In the last case, $\mathrm{typ}\left(U_{C\psi }^{da}\right)\in \left\{\gamma ,\delta \right\}$.*

**Proof.**  Let n∈N and b,c∈{i,d,a}. We now define the value $M=\underline{\max}\left(U_{1},U_{2}\right)$, where $U_{1}=U_{C_{1}\psi_{1}}^{bc}\left(n\right)$, and $U_{2}=U_{C_{2}\psi_{2}}^{bc}\left(n\right)$. Both $U_{1}$ and $U_{2}$ have values from the set {⌀,∞}∪N (see the definitions before Lemma 2). If $U_{1}=U_{2}=⌀$, then M=⌀. If one of $U_{1},U_{2}$ is equal to ⌀ and another one is equal to a number m∈N, then M=m. If $U_{1},U_{2}\in N$, then $M=\max(U_{1},U_{2})$. If at least one of $U_{1},U_{2}$ is equal to *∞*, then M=∞. □

The following equality follows from the definition of the partial function $U_{C\psi }^{bc}\left(n\right)$, where n∈N, and b,c∈{i,d,a}: $U_{C\psi }^{bc}\left(n\right)=\underline{\max}\left(U_{C_{1}\psi_{1}}^{bc}\left(n\right),U_{C_{2}\psi_{2}}^{bc}\left(n\right)\right)$. Later in the proof, we will use this equality without special mention. From this equality, we obtain $\mathrm{typ}\left(U_{C_{1}\psi_{1}}^{bc}\right)⪯\mathrm{typ}\left(U_{C\psi }^{bc}\right)$ and $\mathrm{typ}\left(U_{C_{2}\psi_{2}}^{bc}\right)⪯\mathrm{typ}\left(U_{C\psi }^{bc}\right)$. We now consider two different cases separately: (1) $\mathrm{typ}\left(U_{C_{1}\psi_{1}}^{bc}\right)=\mathrm{typ}\left(U_{C_{2}\psi_{2}}^{bc}\right)$ and (2) $\mathrm{typ}\left(U_{C_{1}\psi_{1}}^{bc}\right)\neq \mathrm{typ}\left(U_{C_{2}\psi_{2}}^{bc}\right)$. Thus, we have the following:

(1) Let $\mathrm{typ}\left(U_{C_{1}\psi_{1}}^{bc}\right)=\mathrm{typ}\left(U_{C_{2}\psi_{2}}^{bc}\right)$.

(a) Let $\mathrm{typ}\left(U_{C_{1}\psi_{1}}^{bc}\right)=\mathrm{typ}\left(U_{C_{2}\psi_{2}}^{bc}\right)=\alpha$. Since the functions $U_{C_{1}\psi_{1}}^{bc}$ and $U_{C_{2}\psi_{2}}^{bc}$ are both bounded from above, we obtain that the function $U_{C\psi }^{bc}=\underline{\max}\left(U_{C_{1}\psi_{1}}^{bc},U_{C_{2}\psi_{2}}^{bc}\right)$ is also bounded from above. From this, it follows that $\mathrm{typ}\left(U_{C\psi }^{bc}\right)=\tilde{\max}(\mathrm{typ}\left(U_{C_{1}\psi_{1}}^{bc}\right),$$\mathrm{typ}(U_{C_{2}\psi_{2}}^{bc}))=\alpha$.

(b) Let $\mathrm{typ}\left(U_{C_{1}\psi_{1}}^{bc}\right)=\mathrm{typ}\left(U_{C_{2}\psi_{2}}^{bc}\right)=\beta$. From the fact that $Dom^{+}\left(U_{C_{1}\psi_{1}}^{bc}\right)$ and $Dom^{+}\left(U_{C_{2}\psi_{2}}^{bc}\right)$ are both finite, we obtain that $Dom^{+}\left(U_{C\psi }^{bc}\right)$ is also finite. Similarly, one can show that $U_{C\psi }^{bc}$ is unbounded from above on C. From here, it follows that $\mathrm{typ}\left(U_{C\psi }^{bc}\right)=\tilde{\max}(\mathrm{typ}\left(U_{C_{1}\psi_{1}}^{bc}\right),$$\mathrm{typ}(U_{C_{2}\psi_{2}}^{bc}))=\beta$.

(c) Let $\mathrm{typ}\left(U_{C_{1}\psi_{1}}^{bc}\right)=\mathrm{typ}\left(U_{C_{2}\psi_{2}}^{bc}\right)=\gamma$. From here, it follows that the function $\psi^{b}$ is unbounded from above on C. From Proposition 1, it follows that bc belongs to the set {ii,di,dd,da,ai,ad,aa}. Let c=b. Using Lemma 4, we obtain $\mathrm{typ}(U_{C\psi }^{bb})=\gamma$. Let bc∈{di,ai,ad}. Using Lemma 3 and the inequalities $\mathrm{typ}\left(U_{C_{1}\psi_{1}}^{bc}\right)⪯\mathrm{typ}\left(U_{C\psi }^{bc}\right)$ and $\mathrm{typ}\left(U_{C_{2}\psi_{2}}^{bc}\right)⪯\mathrm{typ}\left(U_{C\psi }^{bc}\right)$, we obtain $\mathrm{typ}(U_{C\psi }^{bc})=\gamma$. The only case left is when bc=da. Since there is no n∈N for which $U_{C_{1}\psi_{1}}^{bc}\left(n\right)=\infty$ or $U_{C_{2}\psi_{2}}^{bc}\left(n\right)=\infty$, then according to Lemma 2, we obtain that $Dom(U_{C\psi }^{bc})$ is an infinite set. Therefore, $\mathrm{typ}(U_{C\psi }^{bc})\neq \epsilon$, and hence, $\mathrm{typ}\left(U_{C\psi }^{bc}\right)\in \left\{\gamma ,\delta \right\}$. From Proposition 6, it follows that both cases are possible. Thus, we have the following:

(d) Let $\mathrm{typ}\left(U_{C_{1}\psi_{1}}^{bc}\right)=\mathrm{typ}\left(U_{C_{2}\psi_{2}}^{bc}\right)=\delta$. From here, it follows that there is no n∈N for which $U_{C_{1}\psi_{1}}^{bc}\left(n\right)=\infty$ or $U_{C_{2}\psi_{2}}^{bc}\left(n\right)=\infty$. Using Lemma 2, we conclude that $Dom(U_{C\psi }^{bc})$ is an infinite set. From the fact that $Dom^{-}\left(U_{C_{1}\psi_{1}}^{bc}\right)$ and $Dom^{-}\left(U_{C_{2}\psi_{2}}^{bc}\right)$ are both finite, we obtain that $Dom^{-}\left(U_{C\psi }^{bc}\right)$ is also finite. Therefore, $\mathrm{typ}\left(U_{C\psi }^{bc}\right)=\tilde{\max}\left(\mathrm{typ}\left(U_{C_{1}\psi_{1}}^{bc}\right),\mathrm{typ}\left(U_{C_{2}\psi_{2}}^{bc}\right)\right)=\delta$.

(e) Let $\mathrm{typ}\left(U_{C_{1}\psi_{1}}^{bc}\right)=\mathrm{typ}\left(U_{C_{2}\psi_{2}}^{bc}\right)=\epsilon$. Since both $Dom(U_{C_{1}\psi_{1}}^{bc})$ and $Dom(U_{C_{2}\psi_{2}}^{bc})$ are finite sets, we obtain that $Dom(U_{C\psi }^{bc})$ is also a finite set. Therefore, $\mathrm{typ}\left(U_{C\psi }^{bc}\right)=\tilde{\max}\left(\mathrm{typ}\left(U_{C_{1}\psi_{1}}^{bc}\right),\mathrm{typ}\left(U_{C_{2}\psi_{2}}^{bc}\right)\right)=\epsilon$.

(2) Let $\mathrm{typ}\left(U_{C_{1}\psi_{1}}^{bc}\right)\neq \mathrm{typ}\left(U_{C_{2}\psi_{2}}^{bc}\right)$. Denote $f=U_{C_{1}\psi_{1}}^{bc}$ and $g=U_{C_{2}\psi_{2}}^{bc}$. Let typ(f)⪯typ(g). We now consider a number of cases.

(a) Let typ(g)=ϵ. From here, it follows that Dom(g) is a finite set. Taking into account this fact, we obtain that $Dom(\underline{\max}\left(f,g\right))$ is also a finite set. Therefore, $\mathrm{typ}\left(\underline{\max}\left(f,g\right)\right)=\tilde{\max}\left(\mathrm{typ}\left(f\right),\mathrm{typ}\left(g\right)\right)=\epsilon$. Later, we assume that typ(g)≠ϵ.

(b) Let typ(f)=α. Then, both *f* and *g* are nondecreasing functions, *f* is bounded from above, and *g* is unbounded from above. From here, it follows that there exists $n_{0}\in N$ such that f(n)<g(n) for any $n\in N,n\geq n_{0}$. Using this fact, we conclude that $\underline{\max}\left(f\left(n\right),g\left(n\right)\right)=g\left(n\right)$ for $n\geq n_{0}$. Therefore, $\mathrm{typ}\left(\underline{\max}\left(f,g\right)\right)=\tilde{\max}\left(\mathrm{typ}\left(f\right),\mathrm{typ}\left(g\right)\right)=\mathrm{typ}\left(g\right)$. Later, we will assume that typ(f)≠α. It means we should only consider the pairs (typ(f),typ(g))∈{(β,δ),(β,γ),(γ,δ)}.

(c) Let typ(f)=β,typ(g)=δ. From here, it follows that $Dom^{-}\left(f\right),Dom^{+}\left(g\right)$ are both infinite sets, and $Dom^{+}\left(f\right),Dom^{-}\left(g\right)$ are both finite sets. Taking into account that both *f* and *g* are nondecreasing functions, we obtain that there exists $n_{0}\in N$ such that f(n)<g(n) for any $n\in N,n\geq n_{0}$. Therefore, $\mathrm{typ}\left(\underline{\max}\left(f,g\right)\right)=\tilde{\max}\left(\mathrm{typ}\left(f\right),\mathrm{typ}\left(g\right)\right)=\mathrm{typ}\left(g\right)=\delta$.

(d) Let typ(f)=β,typ(g)=γ. Then, $Dom^{+}\left(\underline{\max}\left(f,g\right)\right)$ is an infinite set. Taking into account that $Dom^{-}\left(g\right)$ is an infinite set and that $Dom^{+}\left(f\right)$ is a finite set, we obtain that $Dom^{-}\left(\underline{\max}\left(f,g\right)\right)$ is also an infinite set. Therefore, $\mathrm{typ}\left(\underline{\max}\left(f,g\right)\right)=\tilde{\max}\left(\mathrm{typ}\left(f\right),\mathrm{typ}\left(g\right)\right)=\mathrm{typ}\left(g\right)=\gamma$.

(e) Let typ(f)=γ,typ(g)=δ. From here, it follows that $Dom^{+}\left(\underline{\max}\left(f,g\right)\right)$ is an infinite set, and $Dom^{-}\left(\underline{\max}\left(f,g\right)\right)$ is a finite set. Therefore, $\mathrm{typ}\left(\underline{\max}\left(f,g\right)\right)=\tilde{\max}(\mathrm{typ}\left(f\right),$typ(g))=typ(g)=δ. □

The next statement follows immediately from Proposition 1 and Theorem 3.

**Figure 9:**
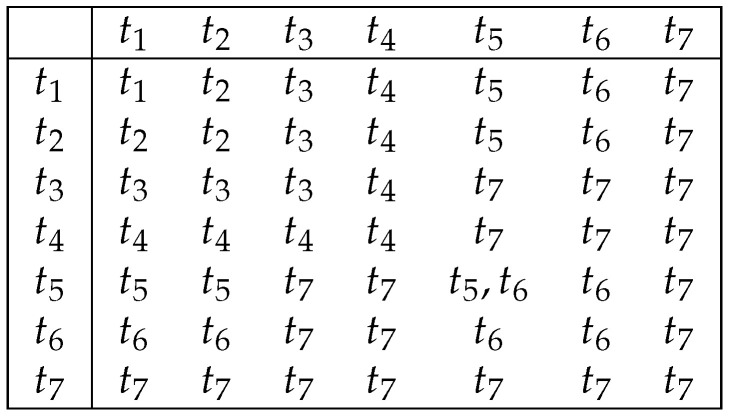
Possible upper types of a union of two compatible t pairs.

**Corollary** **2.** *Let $\tau_{1}$ and $\tau_{2}$ be compatible t pairs, and let τ be a union of these t pairs. Then, the possible values of ${\mathrm{typ}}_{u}\left(\tau \right)$ are in the table shown in Figure 9 in the intersection of the row labeled with ${\mathrm{typ}}_{u}\left(\tau_{1}\right)$ and the column labeled with ${\mathrm{typ}}_{u}\left(\tau_{2}\right)$.*

To finalize the study of unions of t pairs, we prove the following statement:

**Proposition** **6.** *(a) There exist compatible t pairs $\tau_{1}^{1}$ and $\tau_{2}^{1}$ and their union $\tau^{1}$ such that ${\mathrm{typ}}_{u}\left(\tau_{1}^{1}\right)={\mathrm{typ}}_{u}\left(\tau_{2}^{1}\right)={\mathrm{typ}}_{u}\left(\tau^{1}\right)=t_{5}$.*

*(b) There exist compatible t pairs $\tau_{1}^{2}$ and $\tau_{2}^{2}$ and their union $\tau^{2}$ such that ${\mathrm{typ}}_{u}\left(\tau_{1}^{2}\right)={\mathrm{typ}}_{u}\left(\tau_{2}^{2}\right)=t_{5}$ and ${\mathrm{typ}}_{u}\left(\tau^{2}\right)=t_{6}$.*

**Figure 10:**
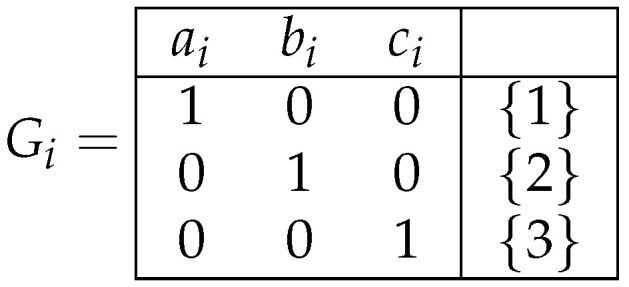
Decision table $G_{i}$.

**Proof.**  For i∈N, we denote $F_{i}=\left\{a_{i},b_{i},c_{i}\right\}$, and $G_{i}$ in the decision table depicted in Figure 10. We study the t pair $\left(T_{i},\psi_{i}\right)$, where $T_{i}$ is the closed class of decision tables from $M_{2}\left(F_{i}\right)$, which is equal to $\left[G_{i}\right]$, and $\psi_{i}$ is a complexity measure over $M_{2}\left(F_{i}\right)$ defined in the following way: $\psi_{i}\left(\lambda \right)=0,\psi_{i}\left(a_{i}\right)=\psi_{i}\left(b_{i}\right)=\psi_{i}\left(c_{i}\right)=i$ and $\psi_{i}\left(\alpha \right)=i+1$ if $\alpha \in F_{i}^{*}$ and |α|≥2. □

We now study the function $U_{T_{i}\psi_{i}}^{da}$. Since the operations of the duplication of columns and the permutation of columns do not change the minimum complexity of the deterministic and nondeterministic decision trees, we only consider the operations of the changing of decisions and the removal of columns.

**Figure 11:**
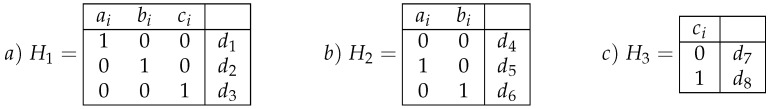
Decision tables from closed class $T_{i}$, where $d_{1},\ldots ,d_{8}\in P\left(N\right)$.

Using these operations, the decision tables from $T_{i}$ can be obtained from $G_{i}$ in three ways: (a) only through the changing of decisions, (b) by removing one column and through the changing of decisions, and (c) by removing two columns and through the changing of decisions. Figure 11 demonstrates examples of the decision tables from $T_{i}$ for each case. Without loss of generality, we can restrict ourselves to considering these three tables: $H_{1}$, $H_{2}$, and $H_{3}$.

We consequently have the following:

(a) There are three different cases for the table $H_{1}$: (i) the sets of decisions $d_{1},d_{2},d_{3}$ are pairwise disjoint, (ii) there are l,t∈{1,2,3} such that $l\neq t,d_{l}\cap d_{t}\neq ⌀$ and $d_{1}\cap d_{2}\cap d_{3}=⌀$, and (iii) $d_{1}\cap d_{2}\cap d_{3}\neq ⌀$. In the first case, $\psi_{i}^{a}\left(H_{1}\right)=i$ and $\psi_{i}^{d}\left(H_{1}\right)=i+1$. In the second case, $\psi_{i}^{a}\left(H_{1}\right)=i$ and $\psi_{i}^{d}\left(H_{1}\right)=i$. In the third case, $\psi_{i}^{a}\left(H_{1}\right)=0$ and $\psi_{i}^{d}\left(H_{1}\right)=0$.

(b) There are three different cases for the table $H_{2}$: (i) the sets of decisions $d_{4},d_{5},d_{6}$ are pairwise disjoint, (ii) there are l,t∈{4,5,6} such that $l\neq t,d_{l}\cap d_{t}\neq ⌀$ and $d_{4}\cap d_{5}\cap d_{6}=⌀$, and (iii) $d_{4}\cap d_{5}\cap d_{6}\neq ⌀$. In the first case, $\psi_{i}^{a}\left(H_{2}\right)=i+1$, and $\psi_{i}^{d}\left(H_{2}\right)=i+1$. In the second case, we have either $\psi_{i}^{a}\left(H_{2}\right)=\psi_{i}^{d}\left(H_{2}\right)=i+1$ or $\psi_{i}^{a}\left(H_{2}\right)=\psi_{i}^{d}\left(H_{2}\right)=i$ depending on the intersecting decision sets. In the third case, $\psi_{i}^{a}\left(H_{2}\right)=0$, and $\psi_{i}^{d}\left(H_{2}\right)=0$.

(c) There are two different cases for the table $H_{3}$: (i) $d_{7}\cap d_{8}=⌀$ and (ii) $d_{7}\cap d_{8}\neq ⌀$. In the first case, $\psi_{i}^{a}\left(H_{3}\right)=i$, and $\psi_{i}^{d}\left(H_{3}\right)=i$. In the second case, $\psi_{i}^{a}\left(H_{3}\right)=0$, and $\psi_{i}^{d}\left(H_{3}\right)=0$.

As a result, we obtain that, for any n∈N,

(3) $$U_{T_{i}\psi_{i}}^{da}\left(n\right)=\left\{\begin{array}{cc} 0, & n<i,\\ i+1, & n\geq i. \end{array}\right.$$

Let *K* be an infinite subset of the set N. Denote $F_{K}=\cup_{i\in K}F_{i}$ and $T_{K}=\cup_{i\in K}\left[G_{i}\right]$. It is clear that $T_{K}$ is a closed class of decision tables from $M_{2}\left(F_{K}\right)$. We now define a complexity measure $\psi_{K}$ over $M_{2}\left(F_{K}\right)$. Let $\alpha \in F_{K}^{*}$. If $\alpha \in F_{i}^{*}$ for some i∈K; then, $\psi_{K}\left(\alpha \right)=\psi_{i}\left(\alpha \right)$. If α contains letters from both $F_{i}$ and $F_{j}$, and if i≠j, then $\psi_{K}\left(\alpha \right)=0$.

Let $K=\{n_{j}:j\in N\}$ and $n_{j}<n_{j+1}$ for any j∈N. We define a function $\varphi_{K}:N\to N$ as follows. Let n∈N. If $n<n_{0}$, then $\varphi_{K}\left(n\right)=0$. Let, for some j∈N, that $n_{j}\leq n<n_{j+1}$. Then, $\varphi_{K}\left(n\right)=n_{j}$. Using (3), one can show that, for any n∈N,

$$U_{T_{K}\psi_{K}}^{da}\left(n\right)=\varphi_{K}\left(n\right).$$

Using this equality, one can prove that $\mathrm{typ}(U_{T_{K}\psi_{K}}^{da})=\gamma$ if the set N∖K is infinite and that $\mathrm{typ}(U_{T_{K}\psi_{K}}^{da})=\delta$ if the set N∖K is finite.

Denote $K_{1}^{1}=\left\{3j:j\in N\right\}$, $K_{2}^{1}=\left\{3j+1:j\in N\right\}$ and $K^{1}=K_{1}^{1}\cup K_{2}^{1}$. Denote $\tau_{1}^{1}=\left(T_{K_{1}^{1}},\psi_{K_{1}^{1}}\right)$, $\tau_{2}^{1}=\left(T_{K_{2}^{1}},\psi_{K_{2}^{1}}\right)$, and $\tau^{1}=\left(T_{K^{1}},\psi_{K^{1}}\right)$. One can show that the t pairs $\tau_{1}^{1}$ and $\tau_{2}^{1}$ are compatible and that $\tau^{1}$ is a union of $\tau_{1}^{1}$ and $\tau_{2}^{1}$. It is easy to prove that $\mathrm{typ}\left(U_{T_{K_{1}^{1}}\psi_{K_{1}^{1}}}^{da}\right)=\mathrm{typ}\left(U_{T_{K_{2}^{1}}\psi_{K_{2}^{1}}}^{da}\right)=\mathrm{typ}\left(U_{T_{K^{1}}\psi_{K^{1}}}^{da}\right)=\gamma$. Using Proposition 2, we obtain ${\mathrm{typ}}_{u}\left(\tau_{1}^{1}\right)={\mathrm{typ}}_{u}\left(\tau_{2}^{1}\right)={\mathrm{typ}}_{u}\left(\tau^{1}\right)=t_{5}$.

Denote $K_{1}^{2}=\left\{2j:j\in N\right\}$, $K_{2}^{2}=\left\{2j+1:j\in N\right\}$ and $K^{2}=K_{1}^{2}\cup K_{2}^{2}=N$. Denote $\tau_{1}^{2}=\left(T_{K_{1}^{2}},\psi_{K_{1}^{2}}\right)$, $\tau_{2}^{2}=\left(T_{K_{2}^{2}},\psi_{K_{2}^{2}}\right)$, and $\tau^{2}=\left(T_{K^{2}},\psi_{K^{2}}\right)$. One can show that the t pairs $\tau_{1}^{2}$ and $\tau_{2}^{2}$ are compatible and that $\tau^{2}$ is a union of $\tau_{1}^{2}$ and $\tau_{2}^{2}$. It is easy to prove that $\mathrm{typ}\left(U_{T_{K_{1}^{2}}\psi_{K_{1}^{2}}}^{da}\right)=\mathrm{typ}\left(U_{T_{K_{2}^{2}}\psi_{K_{2}^{2}}}^{da}\right)=\gamma$ and $\mathrm{typ}(U_{T_{K^{2}}\psi_{K^{2}}}^{da})=\delta$. Using Proposition 2, we obtain ${\mathrm{typ}}_{u}\left(\tau_{1}^{2}\right)={\mathrm{typ}}_{u}\left(\tau_{2}^{2}\right)=t_{5}$ and ${\mathrm{typ}}_{u}\left(\tau^{2}\right)=t_{6}$. □

## 7. Proofs of Theorems 1 and 2

First, we consider some auxiliary statements.

**Definition** **25.** *Let us define a function ρ:{α,β,γ,δ,ϵ}→{α,β,γ,δ,ϵ} as follows: ρ(α)=ϵ,ρ(β)=δ,ρ(γ)=γ,ρ(δ)=β,ρ(ϵ)=α.*

**Proposition** **7** (Proposition 5.1 [22]). *Let X be a nonempty set $f:X\to N,g:X\to N,U^{fg}\left(n\right)=$max{f(x):x∈X,g(x)≤n}, and $L^{gf}\left(n\right)=\min\left\{g\left(x\right):x\in X,f\left(x\right)\geq n\right\}$ for any n∈N. Then, $\mathrm{typ}\left(L^{gf}\right)=\rho \left(\mathrm{typ}\left(U^{fg}\right)\right)$.*

Using Proposition 7, we obtain the following statement:

**Proposition** **8.** *Let (C,ψ) be a t pair, and b,c∈{i,d,a}. Then, $\mathrm{typ}(L_{C\psi }^{cb})=$$\rho (\mathrm{typ}\left(U_{C\psi }^{bc}\right))$.*

**Corollary** **3.** *Let (C,ψ) be a t pair, and i∈{1,…,7}. Then, ${\mathrm{typ}}_{u}\left(C,\psi \right)=t_{i}$ if and only if $\mathrm{typ}\left(C,\psi \right)=T_{i}$.*

**Proof of Theorem** **1.** The statement of the theorem follows from Propositions 1 and 3 and from Corollary 3. □

**Proof of Theorem** **2.** The statement of the theorem follows from Propositions 2 and 4 and from Corollary 3. □

## 8. Conclusions

This paper is devoted to a comparative analysis of the deterministic and nondeterministic decision tree complexity for decision tables from closed classes. It is a qualitative research: we have considered a finite number of types of the behavior of functions characterizing the relationships among different parameters of decision tables. In this paper, we have enumerated all the realizable types of t pairs and limited t pairs. We have also defined the notion of a union of two t pairs and studied the upper type of the resulting t pair, thus depending on the upper types of the initial t pairs. The obtained results allow us to point out cases where the complexity of deterministic and nondeterministic decision trees is essentially less than the complexity of the decision table. Future publications will be related to a quantitative research: we will study the lower and upper bounds on the considered functions.

## Data Availability

Data are contained within the article.
